# 647. Cefepime Versus Carbapenems for the Treatment of ESBL-producing *Enterobacterales* among Non-blood Isolates

**DOI:** 10.1093/ofid/ofac492.699

**Published:** 2022-12-15

**Authors:** Brandon Garcia, Madeline King, Geena Kludjian, Alexandra Hanretty

**Affiliations:** Philadelphia College of Pharmacy, Philadelphia, Pennsylvania; Cooper University Hospital, Camden, New Jersey; Cooper University Hospital, Camden, New Jersey; Cooper University Healthcare, Philadelphia, Pennsylvania

## Abstract

**Background:**

The incidence of organisms with extended-spectrum beta-lactamases (ESBLs) is increasing. The data for using cefepime in ESBL-producing *Enterobacterales* infections is conflicting. More favorable outcomes are likely if minimum inhibitory concentrations (MICs) < 2 mcg/mL. The aim of this study is to compare the efficacy of cefepime versus carbapenems for ESBL-producing, non-bloodstream *Enterobacterales* infections.

**Methods:**

This study was a single-center retrospective cohort study of patients who received cefepime or a carbapenem for at least 72 hours for the definitive treatment of an ESBL-producing *Enterobacterales* non-bloodstream infection between Jan. 1, 2011 and Sept. 30, 2021. ESBL production was identified if the isolate either had a positive confirmatory ESBL test from VITEK 2 (bioMérieux) or phenotypic resistance to ceftriaxone. Isolates had to have a MIC < 2 mcg/mL to cefepime or be susceptible to carbapenems. Isolates with a cefepime MIC 4-8 mcg/mL were not included due to likely higher rates of treatment failure. The primary endpoint was clinical failure, defined as persistence of symptoms requiring escalation of therapy or death. Descriptive statistics were used to compare groups. A univariate analysis and odds ratio was calculated for clinical outcomes.

**Results:**

One hundred patients were included. Twenty-two patients received cefepime and 78 received a carbapenem. Table 1 describes patient characteristics and clinical outcomes. Most patients had a urinary tract infection (UTI) (94%). More patients receiving cefepime were admitted to the intensive care unit (40.9% versus 15.4%). Treatment with cefepime displayed higher rates of clinical failure when compared to treatment with carbapenems (13.6% versus 6.4%). Cohen’s d-test for clinical failure was 0.46, indicating a medium effect.

**Figure:**
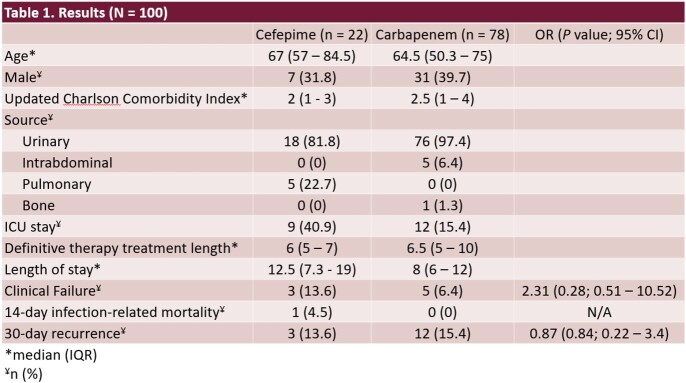

**Conclusion:**

Based on our analysis, cefepime may have higher rates of clinical failure when compared to carbapenem treatment for ESBL-producing *Enterobacterales*. Most of our patients were treated for UTIs and the sample size for both arms were limited, particularly for the cefepime arm. Further research is needed to confirm the role of cefepime as a carbapenem-sparing option in the treatment of these drug-resistant infections.

**Disclosures:**

**Madeline King, PharmD**, Shionogi: Honoraria|Tetraphase: speakers' bureau.

